# Deep Eutectic Solvent-Based Microwave-Assisted Method for Extraction of Hydrophilic and Hydrophobic Components from Radix *Salviae miltiorrhizae*

**DOI:** 10.3390/molecules21101383

**Published:** 2016-10-17

**Authors:** Jue Chen, Mengjun Liu, Qi Wang, Huizhi Du, Liwei Zhang

**Affiliations:** 1 Institute of Molecule Science, Key Laboratory of Chemical Biology and Molecular Engineering of Ministry of Education, Shanxi University, Taiyuan 030006, China; juechenyzm@sina.com (J.C.); 15735170301@163.com (M.L.); 15735158577@163.com (Q.W.); duhuizhi@sxu.edu.cn (H.D.); 2 Modern Research Center for Traditional Chinese Medicine, Shanxi University, Taiyuan 030006, Shanxi, China

**Keywords:** deep eutectic solvents, Radix *Salviae miltiorrhizae*, microwave-assisted extraction, response surface methodology

## Abstract

Deep eutectic solvents (DESs) have attracted significant attention as a promising green media. In this work, twenty-five kinds of benign choline chloride-based DESs with microwave-assisted methods were applied to quickly extract active components from Radix *Salviae miltiorrhizae*. The extraction factors, including temperature, time, power of microwave, and solid/liquid ratio, were investigated systematically by response surface methodology. The hydrophilic and hydrophobic ingredients were extracted simultaneously under the optimized conditions: 20 vol% of water in choline chloride/1,2-propanediol (1:1, molar ratio) as solvent, microwave power of 800 W, temperature at 70 °C, time at 11.11 min, and solid/liquid ratio of 0.007 g·mL^−1^. The extraction yield was comparable to, or even better than, conventional methods with organic solvents. The microstructure alteration of samples before and after extraction was also investigated. The method validation was tested as the linearity of analytes (*r*^2^ > 0.9997 over two orders of magnitude), precision (intra-day relative standard deviation (RSD) < 2.49 and inter-day RSD < 2.96), and accuracy (recoveries ranging from 95.04% to 99.93%). The proposed DESs combined with the microwave-assisted method provided a prominent advantage for fast and efficient extraction of active components, and DESs could be extended as solvents to extract and analyze complex environmental and pharmaceutical samples.

## 1. Introduction

Deep eutectic solvents (DESs) were first reported by Abbott et al. in 2003 [1], which were generally formed by mixing a hydrogen bonding acceptor (HBA) with a hydrogen bonding donor (HBD) after continuous heating and stirring. This eutectic mixture has a much lower melting point than the original HBA and HBD. DESs are always named as ‘ionic liquid analogues’ for their similar properties to ionic liquids, such as eco-friendliness, negligible volatility, and adjustable viscosity [2,3]. Compared with ionic liquids, which are the most popular green solvents [4,5,6], DESs have the advantage of low cost, easy synthesis, biodegradability [7], high solubilization strength [8,9], and low- or even non-toxicity [10,11]. Therefore, DESs become new favorites for scientific research instead of conventional volatile organic solvents and ionic liquids. Until now, DESs as reaction media have been commonly used in the pharmaceutical industry, including sample separation, extraction, and determination [12,13,14,15]. There are considerable references for the application in analytical chemistry of DESs for biological samples [16], metallic elements [17], herbal medicines [18,19,20], nanometer materials [21], and so on. In addition, it is reported that DESs offer advantages in terms of enhancing the stability of active components in herbal medicine [18,22]. Hence, DESs have a great potential as green solvents for simultaneous extraction and determination of different polarity, unstable compounds from traditional Chinese medicine.

Radix *Salviae miltiorrhizae* (named Danshen in Chinese), the roots of *Salvia miltiorrhiza* Bunge from Labiatae, have been mainly used as a traditional medicine for cardiovascular diseases [23,24]. In general, the hydrophilic phenolic acids should be responsible for pharmacological properties, such as anti-platelet activity [25] and free radical scavenging activity [26], and hydrophobic diterpenoid tanshinone has been reported to display diverse pharmacological properties, such as liver-protective [27], anti-cancer [28], and anti-inflammatory effects [29]. Thus, considering the interactions and synergies of Chinese drugs in pharmacology, it is important to simultaneously extract active constituents with different polarities from Radix *Salviae miltiorrhizae* for satisfactory curative effects. The common approaches for the preparation of active ingredients are ultrasonic or heating reflux extraction, using a mixture of water with methanol or ethanol as the solvent [30,31]. However, it is reported that many compounds in Radix *Salviae miltiorrhizae* are unstable in water or ethanol due to the solvent effect and long-time heating [32], which hamper their extraction, analysis, and storage [33,34], and even become a bottleneck in the field of clinical application. Additionally, methanol, as a conventional volatile solvent, has strong toxicity to the human body. Therefore, it is necessary to develop new green solvents with high extraction efficiency, which makes preparation and determination of complex constituents quick and steady.

In this work, active compounds with different polarities from Radix *Salviae miltiorrhizae* were extracted by a microwave-assisted method, and twenty-five kinds of benign choline chloride-based DESs as stabilizing solvent were investigated. Then the effect of water content on extraction efficiency was also studied. The extraction factors (including temperature, time, power of the microwave, and solid/liquid ratio) for five major compounds were optimized systematically by the response surface methodology. The extraction efficiency of the optimal DES-based microwave-assisted method was compared with traditional methods, and the microstructures of Radix *Salviae miltiorrhizae* powders before and after extraction were compared by scanning electron microscopy (SEM).

## 2. Results and Discussion

### 2.1. Effect of Hydrogen Bond Donors of DESs

**Figure 1:**
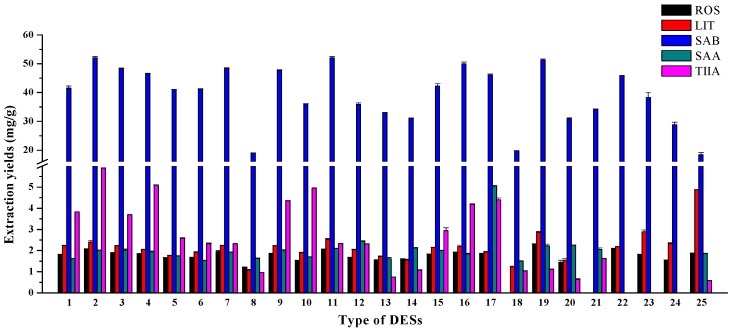
Effect of different types of DESs (temperature = 50.0 °C, time = 10.0 min, microwave power = 800 W, solid/liquid ratio = 0.005 g·mL^−1^).

The structure of hydrogen bond donors (HBDs) has a significant influence on the physicochemical properties of DESs, thus probably affecting the extraction efficiency of rosmarinic acid (ROS), lithospermic acid (LIT), salvionalic acid B (SAB), salvionalic acid A (SAA), and tanshinone ІІA (TІІA). Different HBD were compared including polyhydric alcohols, polyhydric acid, saccharides, and urea. Table 1 listed the abbreviations of the DESs in this work, and the results in Figure 1 showed that DES-2, DES-11, and DES-19 obtained higher yields for extracting SAB than others, DES-25 obtained the highest yield for LIT, and DES-2 obtained the highest yield for TІІA. The difference of extraction amounts for other compounds were less obvious. Hydrogen bond interactions could activate both carbonyl and guanidine groups, which were mainly influenced by DESs components [35]. It is likely that some HBD (such as 1,2-propanediol in DES-2 and urea in DES-25) established the stronger intermolecular hydrogen-bonding interactions with ChCl, which may increase the solubility of targets. Taking into account the extraction efficiency, DES-2 (ChCl-1,2-Propanediol, molar ratio 1:1) was selected for further experimentation.

### 2.2. Effect of the Water Content of DESs

**Figure 2:**
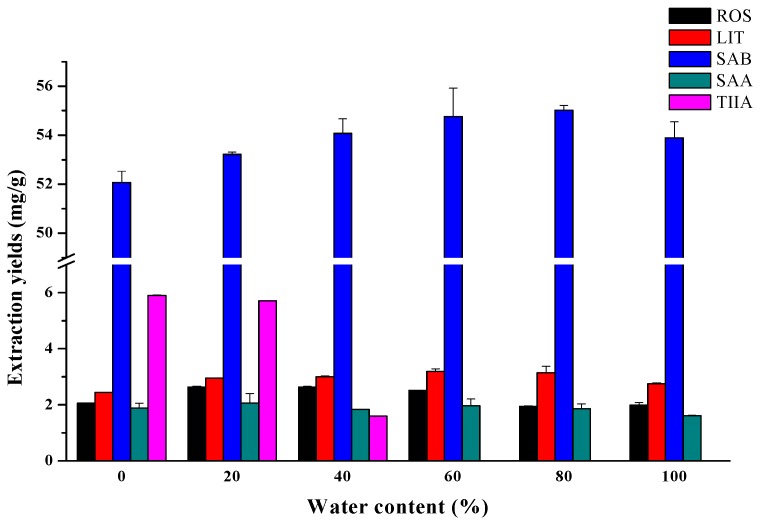
Effect of the water content (temperature = 50.0 °C, time = 10.0 min, microwave power = 800 W, solid/liquid ratio = 0.005 g·mL^−1^).

The large viscosity is one of the disadvantages of DESs, which may influence their penetration in extraction. Adding water to DESs can significantly decrease their viscosity and influence the interactions between the DESs and analytes, which may influence the extraction effects for target compounds [36]. Figure 2 illustrated a formulation containing 0–80 vol% of water in DES-water mixtures, and 100% water was used as the reference solvent. These were investigated at 50 °C to extract five active ingredients for 10 min. In general, the variation trends of the extraction yield for hydrophilic compounds (ROS, LIT, SAB, and SAA) were similar, which were increased first and then decreased with the increase of water content. The amounts of ROS were increased until 60 vol% water content and LIT and SAB were increased until 80 vol%. In contrast, the amounts of hydrophobic compound (TІІA) were highest in pure DESs and decreased with the increase of water content. Therefore, in order to maximize the extraction efficiency of each compound as much as possible, the concentration of 20% water in DES-water mixtures was used as the extraction solvent for further experiments.

### 2.3. Optimization of the Extraction Conditions by RSM

The Design-Expert® software (Version 8.0.6; Stat-Ease Inc., Minneapolis, MN, USA) was applied to optimize a more realistic mode. The preliminary experiments were performed to determine the range of temperature (A, 40–80 °C), time (B, 5–15 min), power of microwave (C, 600–1000 W) and solid/liquid ratio (D, 0.05–0.01 g·mL^−1^). As shown in Table 2, a 29-run Box–Behnken design (BBD) was employed with four variables and three levels, and the mean amounts of corresponding compounds extracted from Radix *Salviae miltiorrhizae* were taken as the responses.

**Figure 3:**
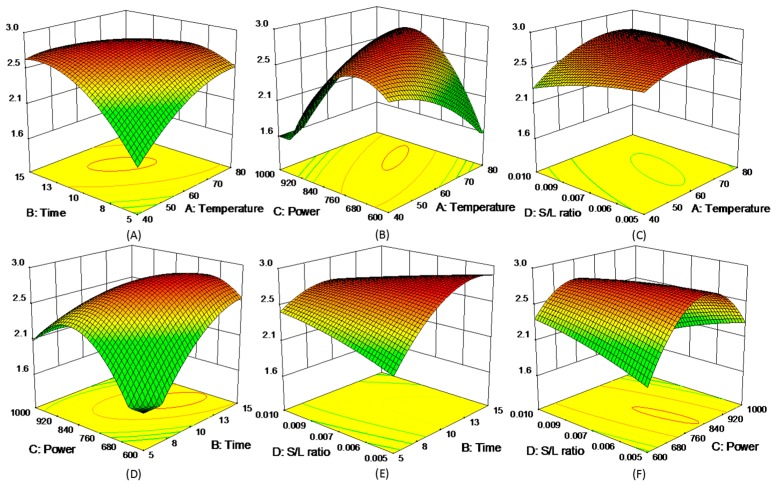
3D response surface plots of ROS.

**Figure 4:**
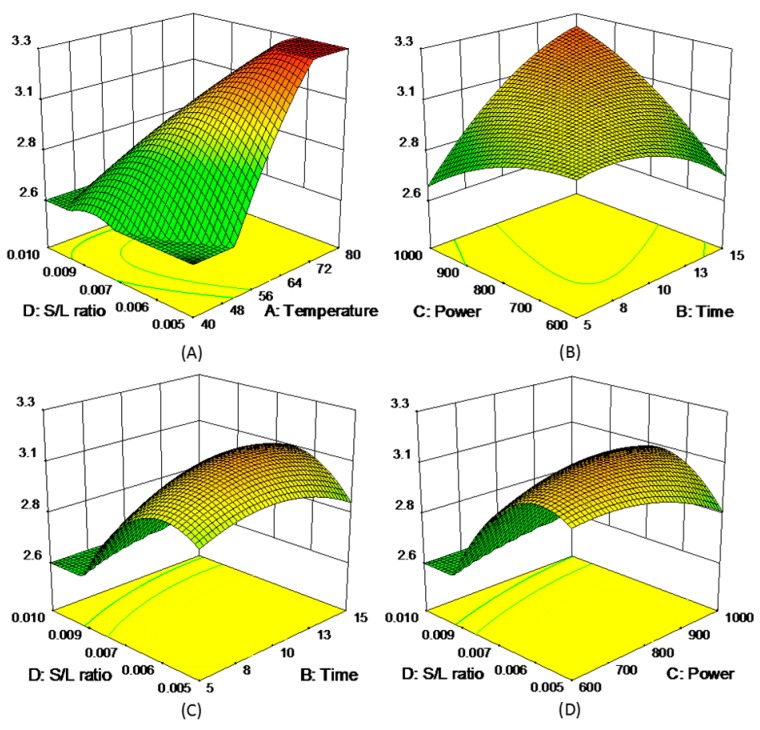
3D response surface plots of LIT.

**Figure 5:**
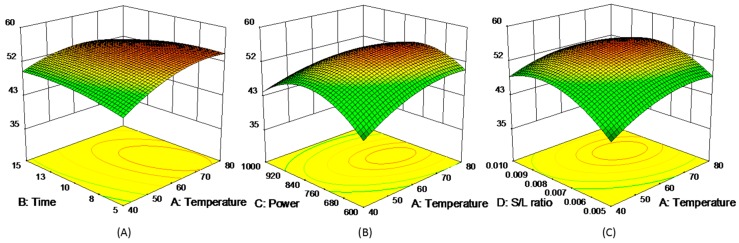
3D response surface plots of SAB.

**Figure 6:**
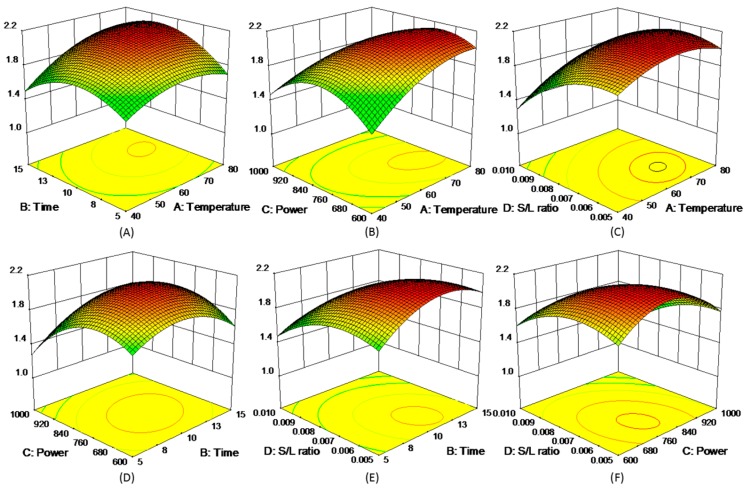
3D response surface plots of SAA.

**Figure 7:**
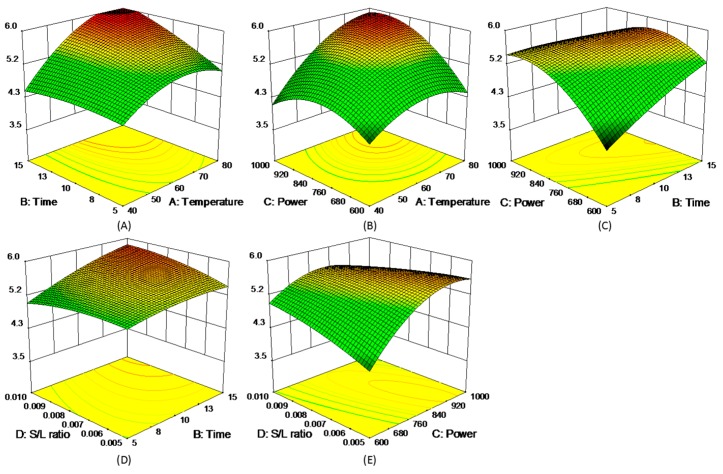
3D response surface plots of TІІA.

The predicted values were obtained from a quadratic model by fitting the experimental data of five responses to the following equations:

*y* = *β_0_* + *β*_1_*A* + *β*_2_*B* + *β*_3_*C* + *β*_4_*D* + *β*_12_*AB* + *β*_13_*AC* + *β*_14_*AD* + *β*_23_*BC* + *β*_24_*BD* + *β*_34_*CD* + *β*_11_A^2^ + *β*_22_*B*^2^ + *β*_33_*C*^2^ + *β*_44_*D*^2^ (1)

where *y* represented each of five experimental responses (*y* = R1, R2, R3, R4, and R5), and the model coefficients (*β*_i_) were summarized in Table S1. The 3D response surfaces of the five responses were schemed in Figure 3, Figure 4, Figure 5, Figure 6 and Figure 7. Figure 3A,D showed the extraction yield of ROS raised by increasing the time. As shown in Figure 3B,F, the extraction yield of ROS improved by increasing the microwave power from 600 to 760 W, and rapidly decreased when exceeding 760 W. Figure 3C,E indicated that the solid/liquid ratio had little effect for ROS. Figure 4A showed that the extraction yield of LIT significantly increased with the increase in temperature, and the other factors had less influence on the extraction yield. For SAB and SAA (Figure 5 and Figure 6), the interaction of factors played an important role for the extraction yield, and when any single factors exceeded a certain value, the extraction yield decreased. Figure 7A–C showed that with the increase of time, temperature, and power, the extraction yield of TІІA increased within a certain range, and the solid/liquid ratio had lesser relationship for extraction efficiency.

Table S2 listed the ANOVA for the quadratic model, and the significance of each coefficient in this model was checked by an F-test and the *P*-value. A *P*-value less than 0.001 indicated that the model terms were significant, and non-significant lacks of fit were good (except ROS). All of the summary statistics demonstrated that the model could be used to navigate the design space to explain the composition effects on the five responses and to select an optimal condition. Finally, the constraints of numerical optimization and the maximization of the desirability function were performed in Table S3 to simultaneously optimize five responses. With these criteria, the extraction conditions (A = 71.01, B = 11.11, C = 827.88, and D = 0.007) for five active ingredients were optimized using the model equation by solving a regression equation. However, based on the limit of the microwave apparatus, we chose the modified condition (A = 70, B = 11.11, C = 800, and D = 0.007) for extracting active compounds, and under these optimized conditions, the extraction amounts of ROS (2.80 mg·g^−1^), LIT (3.19 mg·g^−1^), SAB (53.35 mg·g^−1^), SAA (2.11 mg·g^−1^), and TIIA (5.89 mg·g^−1^) confirmed that the response model was suitable for optimization.

### 2.4. Method Validation

To validate the methodology of the proposed extraction method, the linearity, precision, recovery, and other characteristics were determined by high performance liquid chromatography (HPLC). Table 3 shows that calibration curves of investigated compounds had good linear regressions (*r*^2^ > 0.999) within the test ranges. The limit of quantification (LOQ, based on signal/noise = 10) and the limit of determination (LOD, based on signal/noise = 3) were less than 1.96 µg·mL^−1^ and 0.62 µg·mL^−1^, respectively. The relative standard deviation (RSD) of *intra*-day (*n* = 6) and *inter*-day (*n* = 3) precisions for the peak areas were in the range of 0.15%–1.67% and 0.76%–2.96%, respectively. The extraction recoveries were performed with low (50% standards of the original content), middle (100%), and high (150%) concentrations using proposed pretreatment of Radix *Salviae miltiorrhizae*. Recoveries shown in Table 4 ranged from 95.04% to 99.88% for the five compounds. The validation results suggested that the proposed extraction and analytical methods were reliable.

### 2.5. Comparison of Different Extraction Procedures

In order to evaluate the efficiency of the DES-based microwave-assisted extraction method, different extraction procedures were compared. Three common extraction techniques were selected as follows: microwave-assisted extraction (70 °C, 0.007 g·mL^−1^, 800 W for 11.11 min), ultrasonic-assisted extraction (70 °C, 0.007 g·mL^−1^ for 11.11 min), and heating reflux extraction (80 °C, 0.006 g·mL^−1^, 1 h in Chinese pharmacopoeia) [31]. The extraction yield of the five compounds were determined and listed in Table 5.

Based on the microwave-assisted method, the extraction efficiency of 80% ChCl-PDO (1:1)-20% H_2_O for hydrophilic compounds were obviously more than that of ethanol or different contents of methanol (100%, 75%, and 50% *v*/*v* diluted by water) and, for hydrophobic components, were also much more than that of water. In general, the high extraction efficiency for all of the five target compounds was obtained by 80% ChCl-PDO (1:1)-20% H_2_O, which was comparable to, or even partly better than, 75% methanol. Then, with 80% ChCl-PDO (1:1)-20% H_2_O and 75% methanol as the solvent, respectively, the efficiency of different extraction technologies was also compared. As shown in Table 5, the extraction efficiency was of the following order: microwave-assisted extraction > heating reflux extraction > ultrasonic-assisted extraction, which may be because microwave irradiation could accelerate plant cell rupture and release intracellular products. In addition, the extraction methods of Radix *Salvia miltiorrhiza* in Chinese pharmacopoeia applied two solvents, 75% methanol for SAB and methanol for TIIA, respectively [31]. However, the extraction yield of SAB (53.35 mg·g^−1^) by the DES-based microwave-assisted method was preferable than the method in the Chinese pharmacopoeia (49.31 mg·g^−1^), and the yield of TIIA (5.89 mg·g^−1^) by the DES-based microwave-assisted method was only 1.35% lower than the method in the Chinese pharmacopoeia (5.97 mg·g^−1^), which was within the error range. This might be because longer extraction duration of heating reflux extraction might induce changes in the structure of the target compounds [37]. Therefore, the developed DES-based microwave-assisted extraction could be a more rapid and effective extraction method instead of the method used in the Chinese pharmacopoeia.

### 2.6. Microstructure Alteration of Different Extraction Procedures

**Figure 8:**
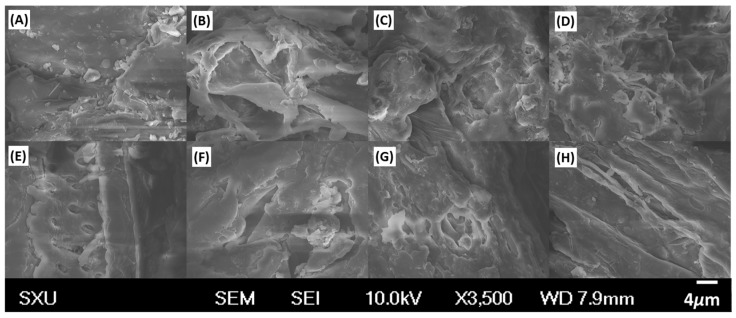
SEM graphics of Radix *Salviae miltiorrhizae* samples. (**A**) Raw materials, (**B**) extracted by DES-based microwave-assisted, (**C**) 75% methanol-based microwave-assisted extraction, (**D**) water-based microwave-assisted extraction, (**E**) DES-based ultrasound-assisted treatment. (**F**) 75% methanol-based ultrasound-assisted extraction, (**G**) DES-based hot reflux-assisted extraction, and (**H**) 75% methanol-based hot reflux-assisted extraction.

In order to illuminate the microstructure alteration during the different extraction procedures, the raw and extracted Radix *Salviae miltiorrhizae* samples were examined by SEM (shown in Figure 8). In the raw sample (Figure 8A). There was no apparent disruption on the cell surface. While under microwave-assisted extraction (11.11 min only), plant cells were already thoroughly ruptured and collapsed in different solvents, and the disruption degree was of the following order: DESs (Figure 8B) > 75% methanol (Figure 8C) > water (Figure 8D). The results indicated that the plant cell easily disrupted in DESs during microwave-assisted treatment, which was conducive to the release of the targets to the extraction solution. The microstructures of the sample were partially destructed under ultrasonic treatment, and a few of the significant ruptures showed on the cell surface (Figure 8E,F). In long-time hot reflux extraction, the sample cells were only slightly destroyed (Figure 8G,H), thus, the analytes were extracted mainly by permeation and solubilization. The results of microstructure alteration coincided with the data of extraction efficiency, which demonstrated that the cell disruption also played an important role in extraction.

## 3. Experimental

### 3.1. Chemicals

EG, PDO, GL, and BDO were purchased from Tianjin Dengfeng Chemical Reagent Factory (Tianjin, China) with purity > 98.0%. OA, LA, SA, MA, MaA, and CA were supplied by Chengdu Kelong Chemical Reagent Factory (Chendu，China) with purity > 99.0%. Mal. Fru, Glu and U were obtained from Beijing Solarbio Science and Technology Co., Ltd. (Beijing, China) with purity > 98.0%. ChCl was obtained from Luye Pharma Group Co., Ltd. (Yantai, China) with purity > 98.0%. ROS (purity > 99.0%), LIT (purity > 98.0%), SAB (purity > 98.0%), SAA (purity > 99.0%), and TІІA (purity > 99.0%) were from Chengdu Must Bio Technology Co., Ltd. (Sichuan, China). Acetonitrile, acetic acid, methanol, ethanol, and other organic solvents of HPLC grade were from MREDA (MREDA Technology Inc., Beijing, China). Water was deionized.

### 3.2. HPLC Analysis

An Agilent 1260 HPLC system (Agilent Technologies, Waldbronn, Germany) equipped with an online degasser, a G1311C quaternary-pump, G1329B auto-samplers, a G1314B VWD detector with wavelength of 280 nm for SAB and 270 nm for TІІA, and a GT-30 column temperature controller maintained at 50 °C was used. HPLC analysis was performed on a Venusil XBP-C18 reversed-phase column (4.6 mm × 250 mm, 5 μm, 100 Å, Agela Technologies, Wilmington, DE, USA). The mobile phase consisted of 0.4% acetic acid-water (A) and acetonitrile (B) in a linear gradient elution of 10%–25% B at 0–10 min, 25%–35% B at 10–20 min, 35%–75% B at 20–25 min, 75%–85% B at 25–40 min, and 85%–100% B at 40–50 min. The flow rate was 1.0 mL·min^−1^ and the sample injection volume was 10 μL. All samples were filtered through 0.45 μm cellulose membranes prior to HPLC analysis. Acquisition and analysis of data were performed by Agilent OpenLAB CDS Chemstation edition Software Ver. C. 01. 07 (Agilent Technologies, Waldbronn, Germany).

### 3.3. Preparation of DESs

All DESs were prepared by the method used in a previous report [37].

### 3.4. Preparation of Herbal Samples

Microwave-based extraction experiments were performed in a microwave oven (XH-100H, Beijing Xianghu Science and Technology Development Co., LTD, Beijing, China) with a temperature probe and power controller. 0.05 g powder of Radix *Salviae miltiorrhizae*, and 10 mL of DESs were put into a microwave reaction flask (50 mL). Then the flask was placed into the microwave extractor for 10 min at 50 °C, with microwave power of 800 W. The solutions were filtered through 0.45 μm filters for HPLC analysis. All of the experiments were performed in triplicate.

## 4. Conclusions

Eco-friendliness and low cost of DESs exhibited good potential as green solvents for the extraction and determination of environmental and pharmaceutical matrices. In our experiment, twenty-five kinds of benign choline chloride-based DESs with different HBDs were used for extracting both hydrophilic and hydrophobic ingredients from Radix *Salviae miltiorrhizae*. By optimization of the HBD and water content, ChCl/1,2-propanediol (1:1, molar ratio) with 20 vol% water was selected to be the best solvent for the extraction of active compounds. According to the BBD test, the extraction yield of analytes were improved under the optimal performance, including a temperature of 70 °C, time of 11.11 min, microwave power of 800 W, and a solid/liquid ratio of 0.007 g·mL^−1^. The microstructure alteration of different extraction procedures testified that the cell disruption also affected the extraction efficiency. In addition, compared to traditional procedures, the optimal extraction method provides unique advantages in terms of lower toxicity, shorter time, and high extraction yield. Therefore, DESs as green solvents can be extended to apply to environmental and pharmaceutical analysis. However, the recovery of DES and the purification of the samples were still challenging, and we need to continue to explore this in follow-up studies.

## Figures and Tables

**Table 1:** Different composition of DESs applied in this work.

No.	Type of HBD	Abbreviation	ChCl/HBD Ratio
DES-1	Ethylene glycol	EG	1:2
DES-2	1,2-Propanediol	PDO	1:1
DES-3			1:2
DES-4			1:3
DES-5	Glycerol	GL	1:1
DES-6			1:2
DES-7			1:3
DES-8			1:4
DES-9	1,4-Butanediol	BDO	1:2
DES-10			1:4
DES-11	Oxalic acid	OA	2:1
DES-12			1:1
DES-13	Succinic acid	SA	2:1
DES-14			1:1
DES-15	Lactic acid	LA	1:1
DES-16	Malonic acid	MaA	1:1
DES-17			1:2
DES-18	Malic acid	MA	1:1
DES-19	Citric acid	CA	2:1
DES-20	Maltose	Mal	1:1
DES-21	Fructose	Fru	2:1
DES-22	Glucose	Glu	2:1
DES-23	Urea	U	2:1
DES-24			1:1
DES-25			1:2

**Table 2:** Box–Behnken design with independent variables and measured responses.

Run	A	B	C	D	ROS	LIT	SAB	SAA	TIIA
1	60	10	800	0.0075	2.78	3.19	55.64	2.12	5.79
2	60	15	1000	0.0075	1.31	3.19	45.82	1.55	5.13
3	60	10	800	0.0075	2.77	3.03	53.62	2.10	5.54
4	60	10	800	0.0075	2.83	3.05	53.48	2.08	5.52
5	40	10	800	0.0100	2.21	2.61	48.88	1.34	4.58
6	40	15	800	0.0075	2.65	2.49	48.74	1.56	4.68
7	60	15	800	0.0100	2.49	2.55	51.24	1.47	5.90
8	60	10	800	0.0075	2.80	2.99	53.29	2.09	5.45
9	40	5	800	0.0075	1.77	2.41	45.84	1.56	4.75
10	60	5	800	0.0050	1.89	2.91	47.16	1.70	5.16
11	60	10	600	0.0100	2.30	2.12	43.98	1.56	4.71
12	60	10	1000	0.0050	2.36	2.92	39.41	1.82	5.78
13	80	10	1000	0.0075	2.59	2.94	45.88	1.58	5.95
14	40	10	800	0.0050	2.69	1.99	41.36	1.85	4.34
15	80	10	800	0.0050	2.60	3.35	47.71	1.94	5.45
16	80	5	800	0.0075	2.58	2.83	52.66	1.64	4.82
17	60	10	600	0.0050	2.08	3.07	41.66	1.88	4.17
18	80	10	800	0.0100	2.61	2.04	50.45	1.76	5.80
19	60	15	600	0.0075	2.64	2.63	47.12	1.54	5.25
20	60	10	1000	0.0100	1.56	2.54	44.77	1.05	5.10
21	80	10	600	0.0075	1.46	2.94	50.65	2.07	4.68
22	40	10	600	0.0075	2.36	2.49	42.21	1.44	4.12
23	80	15	800	0.0075	2.27	3.19	49.92	2.02	5.92
24	60	5	800	0.0100	2.61	2.08	50.03	1.53	4.97
25	60	5	600	0.0075	1.22	3.02	48.63	1.70	4.10
26	60	15	800	0.0050	2.71	2.96	47.02	1.95	5.45
27	60	10	800	0.0075	2.82	3.06	53.39	2.02	5.47
28	40	10	1000	0.0075	1.21	2.60	44.91	1.43	3.97
29	60	5	1000	0.0075	1.95	2.77	46.42	1.34	5.37

A: temperature (°C), B: time (min), C: power (W), D: solid/liquid ratio (g·mL^−1^).

**Table 3:** Calibration curves, test range, LODs, and precisions for analytes by HPLC.

No.	Calibration Curve	*r*^2^	Linear Range	LOQs	LODs	Precision (RSD)		Concentrations
			(μg·mL^−1^)	(μg·mL^−1^)	(μg·mL^−1^)	*Intra-* (*n* = 6)	*Inter-* (*n* = 3)	(mg·g^−1^)
ROS	*y* = 2831.5*x* − 46.721	0.9998	1.61–250.00	0.80	0.24	1.61	2.02	2.80
LIT	*y* = 1442.2*x* − 12.717	0.9999	3.05–350.00	1.37	0.49	1.67	2.96	3.19
SAB	*y* = 1188.8*x* − 40.956	0.9998	2.03–4000.00	1.96	0.62	1.09	1.03	53.35
SAA	*y* = 2907.2*x* − 73.327	0.9997	1.96–195.00	0.87	0.31	2.49	2.83	2.11
TІІA	*y* = 1755.2*x* − 5.950	0.9999	1.96–500.00	1.45	0.48	0.15	0.76	5.89

**Table 4:** Extraction recoveries of the five analytes.

No.	Original/mg	Recovery (Low Spiked)			Recovery (Middle Spiked)			Recovery (High Spiked)		
		Spiked (mg)	Found (mg)	Recovery (%)	Spiked (mg)	Found (mg)	Recovery (%)	Spiked (mg)	Found (mg)	Recovery (%)
1	1.40	0.65	1.97	96.10	1.40	2.78	99.29	2.10	3.40	97.14
2	1.61	0.79	2.29	95.42	1.60	3.19	99.53	2.39	3.95	98.69
3	26.68	13.37	39.76	99.28	26.68	52.15	97.74	40.01	66.61	99.88
4	1.06	0.52	1.51	95.57	1.06	2.01	95.04	1.58	2.57	97.26
5	2.95	1.47	4.40	99.55	2.95	5.69	96.52	4.42	7.36	99.93

**Table 5:** Extraction contents of the five analytes using various extraction procedures.

Extraction Method	Solvent	Extraction Yields (mg·g^−1^)				
		ROS	LIT	SAB	SAA	TIIA
Microwave	80% ChCl-PDO (1:1)/20% H_2_O	2.89 ± 0.03	3.19 ± 0.05	53.35 ± 0.01	2.11 ± 0.01	5.89 ± 0.02
	100% Ethanol	1.89 ± 0.02	2.32 ± 0.07	45.37 ± 0.65	1.69 ± 0.01	4.78 ± 0.00
	100% Methanol	2.06 ± 0.02	2.21 ± 0.02	46.68 ± 0.57	1.87 ± 0.01	5.97 ± 0.05
	75% Methanol	2.75 ± 0.01	2.68 ± 0.02	52.63 ± 0.07	2.54 ± 0.00	5.94 ± 0.05
	50% Methanol	2.80 ± 0.02	2.92 ± 0.04	48.66 ± 0.04	1.69 ± 0.05	3.47 ± 0.08
	Water	2.99 ± 0.02	3.16 ± 0.02	53.89 ± 0.57	1.61 ± 0.01	ND
Ultrasound	80% ChCl-PDO (1:1)/20% H_2_O	1.80 ± 0.01	1.66 ± 0.03	40.81 ± 0.08	1.97 ± 0.03	4.48 ± 0.04
	75% Methanol	0.89 ± 0.02	1.31 ± 0.09	31.39 ± 0.04	1.50 ± 0.02	3.87 ± 0.03
Hot reflux	80% ChCl-PDO (1:1)/20% H_2_O	2.38 ± 0.08	2.78 ± 0.06	41.78 ± 0.02	2.09 ± 0.07	4.98 ± 0.08
	75% Methanol	2.73 ± 0.08	2.93 ± 0.03	49.31 ± 0.09	2.09 ± 0.05	5.25 ± 0.08
	100% Methanol	2.05 ± 0.03	2.12 ± 0.01	43.35 ± 0.21	1.88 ± 0.01	5.97 ± 0.09

ND means not determined because of trace content.

## Supplementary Materials

The following are available online at http://www.mdpi.com/1420-3049/21/10/1383/s1.
