# Supplementary material for: Deep Eutectic Solvent-Based Microwave-Assisted Method for Extraction of Hydrophilic and Hydrophobic Components from Radix Salviae miltiorrhizae
Source: Molecules. 2016 Oct 17;21(10):1383. doi: 10.3390/molecules21101383 (PMC6274102; doi:10.3390/molecules21101383)
Supplement: Supplementary file 1 [file molecules-21-01383-s001.pdf]

Supplementary Materials: Deep Eutectic Solvent-Based Microwave-Assisted Method for Extraction of Hydrophilic and Hydrophobic Components from Radix *Salviae miltiorrhizae*

Jue Chen, Mengjun Liu, Qi Wang, Huizhi Du and Liwei Zhang

Table S1. The results of complete or reduced special-quartic mixture models fit to six responses.

Model Term in Pseudo-Components	Model Coefficients(β_i) Estimated from Data [a]				
	**ROS**	**LIT**	**SAB**	**SAA**	**TIIA**
β_0	2.76	3.06	53.88	2.08	5.55
A-Temp	0.10	0.22	2.11	0.15	0.52
B-Time	0.17	0.08	−0.07	0.05	0.26
C-Power	−0.09	0.06	−0.59	−0.12	0.36
D-S/L ratio	−0.05	−0.27	2.09	−0.20	0.06
AB	−0.30	0	−1.41	0.10	0.29
AC	0.57	0	−1.87	−0.12	0.36
AD	0.12	−0.48	−1.20	0.08	0
BC	−0.51	0.20	0	0.09	−0.35
BD	−0.23	0.10	0	−0.08	0.16
CD	−0.25	0.14	0	−0.11	−0.31
A^2	−0.19	−0.24	−2.56	−0.15	−0.40
B^2	−0.30	−0.10	−1.14	−0.23	−0.10
C^2	−0.68	−0.07	−6.03	−0.31	−0.49
D^2	0	−0.33	−4.50	−0.20	−0.11
Summary statistics					
R-Squared	0.965	0.958	0.957	0.969	0.950
Adj R-Squared	0.934	0.927	0.929	0.938	0.907

[a] Coefficient was statistically significant with at least 95% confidence.

Table S2. The ANOVA for the experimental results of the BBD.

**Source**	**ROS**		**LIT**		**SAB**		**SAA**		**TIIA**	
	**F**	**P**	**F**	**P**	**F**	**P**	**F**	**P**	**F**	**P**
Model	31.65	< 0.0001 [S]	30.48	< 0.0001 [S]	34.23	< 0.0001 [S]	31.38	< 0.0001 [S]	22.04	< 0.0001 [S]
A-Temp	7.05	0.0180 [S]	58.97	< 0.0001 [S]	43.97	< 0.0001 [S]	57.74	< 0.0001 [S]	94.92	< 0.0001 [S]
B-Time	19.90	0.0005 [S]	7.93	0.0124 [S]	0.05	0.8205 [NS]	6.63	0.0221 [S]	24.82	0.0002 [S]
C-Power	5.52	0.0329 [S]	3.85	0.0673 [NS]	3.40	0.0828 [NS]	34.76	< 0.0001 [S]	45.31	< 0.0001 [S]
D-S/L ratio	1.43	0.2499 [NS]	85.97	< 0.0001 [S]	42.94	< 0.0001 [S]	101.80	< 0.0001 [S]	1.25	0.2806 [NS]
AB	20.12	0.0004 [S]	-	-	6.54	0.0204 [S]	7.47	0.0162 [S]	10.21	0.0060 [S]
AC	73.85	< 0.0001 [S]	-	-	11.47	0.0035 [S]	11.92	0.0039 [S]	15.03	0.0015 [S]
AD	3.41	0.0846 [NS]	90.40	< 0.0001 [S]	4.70	0.0447 [S]	5.63	0.0325 [S]	-	-
BC	60.29	< 0.0001 [S]	15.92	0.0011 [S]	-	-	7.08	0.0186 [S]	14.41	0.0018 [S]
BD	12.55	0.0030 [S]	4.28	0.0551 [NS]	-	-	4.97	0.0427 [S]	3.05	0.1010 [NS]
CD	14.78	0.0016 [S]	7.88	0.0126 [S]	-	-	10.47	0.0060 [S]	11.10	0.0046 [S]
A^2	13.18	0.0025 [S]	36.37	< 0.0001 [S]	34.97	< 0.0001 [S]	31.79	< 0.0001 [S]	31.07	< 0.0001 [S]
B^2	34.28	< 0.0001 [S]	5.87	0.0276 [S]	6.89	0.0177 [S]	72.64	< 0.0001 [S]	1.77	0.2028 [NS]
C^2	177.14	< 0.0001 [S]	3.23	0.0914 [NS]	193.92	< 0.0001 [S]	127.03	< 0.0001 [S]	46.83	< 0.0001 [S]
D^2			69.75	< 0.0001 [S]	108.17	< 0.0001 [S]	51.77	< 0.0001 [S]	2.21	0.1574 [NS]
Lack of Fit	36.55	0.0017 [S]	2.08	0.2497 [NS]	1.32	0.4299 [NS]	4.37	0.0842 [S]	2.08	0.2507 [NS]

F: Test statistic, P: *p*-value, LOF: Lack of Fit. [S] means significant, [NS] means not significant.

Table S3. The constraints and results of numerical optimization with the desirability function.

Parameter	Goal	Experimental Region		Weight		Importance	Solutions
		Lower	Upper	Lower	Upper		
Temperature	In range	40	80	1	1	3	71.01
Time	In range	5	15	1	1	3	11.11
Power	In range	600	1000	1	1	3	827.88
S/L ratio	In range	0.005	0.01	1	1	3	0.007
ROS	Maximize	1.21	2.83	1	1	5	2.80
LIT	Maximize	1.99	3.35	1	1	5	3.24
SAB	Maximize	39.41	55.64	1	1	5	53.14
SAA	Maximize	1.05	2.12	1	1	5	2.13
TIIA	Maximize	3.97	5.95	1	1	5	5.85
Desirability							0.94
